# Validation and performance of a multiplex serology assay to quantify antibody responses following SARS‐CoV‐2 infection or vaccination

**DOI:** 10.1002/cti2.1385

**Published:** 2022-04-26

**Authors:** Deidre Wilkins, Anastasia A Aksyuk, Alexey Ruzin, Kevin M Tuffy, Tina Green, Rebecca Greway, Brittany Fikes, Cyrille J Bonhomme, Mark T Esser, Elizabeth J Kelly

**Affiliations:** ^1^ Translational Medicine, Vaccines and Immune Therapies BioPharmaceuticals Medical AstraZeneca Gaithersburg MD USA; ^2^ PPD® Laboratories Vaccine Sciences Lab Richmond VA USA

**Keywords:** antibodies, COVID‐19, electrochemiluminescence, immunoassay, SARS‐CoV‐2, serology

## Abstract

**Objectives:**

Robust, quantitative serology assays are required to accurately measure antibody levels following vaccination and natural infection. We present validation of a quantitative, multiplex, SARS‐CoV‐2, electrochemiluminescent (ECL) serology assay; show correlation with two established SARS‐CoV‐2 immunoassays; and present calibration results for two SARS‐CoV‐2 reference standards.

**Methods:**

Precision, dilutional linearity, ruggedness, analytical sensitivity and specificity were evaluated. Clinical sensitivity and specificity were assessed using serum from prepandemic and SARS‐CoV‐2 polymerase chain reaction (PCR)‐positive patient samples. Assay concordance to the established Roche Elecsys® Anti‐SARS‐CoV‐2 immunoassay and a live‐virus microneutralisation (MN) assay was evaluated.

**Results:**

Standard curves demonstrated the assay can quantify SARS‐CoV‐2 antibody levels over a broad range. Assay precision (10.2−15.1% variability), dilutional linearity (≤ 1.16‐fold bias per 10‐fold increase in dilution), ruggedness (0.89−1.18 overall fold difference), relative accuracy (107−118%) and robust selectivity (102−104%) were demonstrated. Analytical sensitivity was 7, 13 and 7 arbitrary units mL^−1^ for SARS‐CoV‐2 spike (S), receptor‐binding domain (RBD) and nucleocapsid (N) antigens, respectively. For all antigens, analytical specificity was > 90% and clinical specificity was 99.0%. Clinical sensitivities for S, RBD and N antigens were 100%, 98.8% and 84.9%, respectively. Comparison with the Elecsys® immunoassay showed ≥ 87.7% agreement and linear correlation (Pearson *r* of 0.85, *P* < 0.0001) relative to the MN assay. Conversion factors for the WHO International Standard and Meso Scale Discovery® Reference Standard are presented.

**Conclusions:**

The multiplex SARS‐CoV‐2 ECL serology assay is suitable for efficient, reproducible measurement of antibodies to SARS‐CoV‐2 antigens in human sera, supporting its use in clinical trials and sero‐epidemiology studies.

## Graphical abstract

We present validation of a quantitative, multiplex, SARS‐CoV‐2, electrochemiluminescent (ECL) serology assay; show correlation with two established SARS‐CoV‐2 immunoassays; and present calibration results for two SARS‐CoV‐2 reference standards. This assay is suitable for efficient, reproducible measurement of antibodies to SARS‐CoV‐2 antigens in human sera, supporting its use in clinical trials and sero‐epidemiology studies.

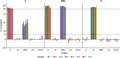

## Introduction

Severe acute respiratory syndrome coronavirus 2 (SARS‐CoV‐2), an enveloped, positive‐sense RNA virus, was identified as the causative agent of coronavirus disease 2019 (COVID‐19) in January 2020.^1^, ^2^ SARS‐CoV‐2 codes for various structural proteins, including the highly immunogenic spike protein (S) that forms characteristic club‐like spike projections from its surface, and the nucleocapsid protein (N) that plays a key role in transcription and viral assembly.^3^, ^4^, ^5^ At the time of manuscript preparation, there have been more than 445 million confirmed cases of COVID‐19 worldwide, and approximately six million people are known to have died from COVID‐19.^6^

In the months following declaration of the pandemic, there was a rapid increase in the development and availability of SARS‐CoV‐2 diagnostics, which was followed by the development and rollout of vaccines and monoclonal antibodies (mAbs) against SARS‐CoV‐2.^4^, ^7^, ^8^, ^9^, ^10^ To facilitate the development of vaccines and therapeutic antibodies and to enable large sero‐epidemiology studies, robust serology assays are needed. A robust anti‐SARS‐CoV‐2 serology assay should ideally: (1) differentiate SARS‐CoV‐2 antibodies from cross‐reactive antibodies (e.g. antibodies to other coronaviruses), (2) differentiate antibody responses following vaccination from those following natural infection, (3) be high‐throughput and easy to perform, (4) reproducibly quantify antibody levels over time (months and years of follow‐up) and (5) be accurate so that results can be compared to other clinical studies and be used to determine correlates of protection.^11^

Quantitative, multiplex electrochemiluminescence (ECL)‐based serology assays allow for sensitive, high‐throughput and simultaneous quantification of immunoglobulin G (IgG) levels to multiple antigens and have been shown to correlate with SARS‐CoV‐2 neutralisation assays.^12^, ^13^, ^14^ Such assays are highly scalable, optimal for complex matrices (typically yielding low interference from serum or plasma),^12^ have a broad dynamic range and have the capacity to multiplex within a single well with small sample volume requirements. The multiplex SARS‐CoV‐2 ECL serology assay presented here is based on Meso Scale Discovery® (MSD) technology (MSD, Rockville, MD, USA); it is a quantitative ECL assay^14^ that uses disposable multi‐spot microtiter plates coated with S, receptor‐binding domain (RBD) and N antigens to detect SARS‐CoV‐2‐specific antibodies present in serum samples.^15^

In this report, we provide an overview of the strategy and validation results for the multiplex SARS‐CoV‐2 ECL serology assay and demonstrate the assay's applicability to complement clinical diagnostics. We also correlate the results of this assay with those from the Food and Drug Administration (FDA)‐authorised Elecsys® Anti‐SARS‐CoV‐2 immunoassay (Roche Diagnostics, Basel, Switzerland) and with a live‐virus microneutralisation (MN) assay developed and validated by the Battelle Biomedical Research Center (BBRC, Columbus, OH, USA). Lastly, we present the assay calibration and potency results of the multiplex SARS‐CoV‐2 ECL serology assay to the World Health Organization (WHO) International Standard and another commonly used calibration standard.

## Results

### Standard curve characterisation and assay validation

#### Standard curve characterisation

**Figure 1:**
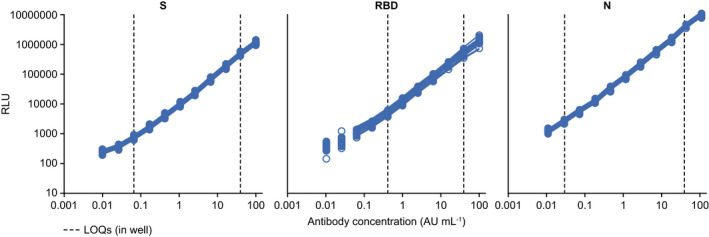
Standard curve precision profiles for SARS‐CoV‐2‐specific S, RBD and N antibodies 11‐point dilution series tested 45 times. AU, arbitrary units; LOQ, limit of quantitation; N, nucleocapsid protein; RBD, receptor‐binding domain; RLU, relative light unit; S, spike protein; SARS‐CoV‐2, severe acute respiratory syndrome coronavirus 2.

A high‐titre, pooled, human serum, collected from polymerase chain reaction (PCR)‐confirmed SARS‐CoV‐2‐positive convalescent samples (see Methods), was used to create an 11‐point reference standard curve for each antigen with concentrations ranging from 0.01 to 100 arbitrary units (AU) mL^−1^. The ECL signals spanned four logs (Figure 1), and 11 standard curve points were used for the SARS‐CoV‐2 S and N antigens and nine points for the RBD antigen. Per the prespecified standard curve criteria, the total number of valid standard curve points for SARS‐CoV‐2 S and N antigens was ≥ 9 and RBD antigen was ≥ 7, and all 45 plates used in the validation met these criteria (Figure 1).

#### Assay precision, dilutional linearity and ruggedness

Intermediate assay precision was determined to be 15.1%, 10.2% and 14.9% geometric coefficient of variance (GCV) for SARS‐CoV‐2 S, RBD and N antigens, respectively (Table 1). Dilutional linearity bias ranged from 1.07‐ to 1.16‐fold per 10‐fold increase in dilution across the three antigens, and ruggedness between the five analysts and two plate lots ranged from 0.89‐ to 1.18‐fold (Table 1). Based on the intermediate precision of the assay, it was determined that a 1.82‐, 1.51‐ and 1.81‐fold increase in antibody levels for S, RBD and N, respectively, were statistically significant (Table 1).

**Table 1:** Multiplex SARS‐CoV‐2 ECL serology assay validation summary characteristics

Assay characteristic	SARS‐CoV‐2 antigen		
	S	RBD	N
LLOQ (AU mL^−1^)			
1:500	33	204	14
1:5000	330	2040	140
1:50 000	3300	20 400	1400
ULOQ (AU mL^−1^)			
1:500	20 000	20 000	20 000
1:5000	200 000	200 000	200 000
1:50 000	2 000 000	2 000 000	2 000 000
LOD (AU mL^−1^)	7	13	7
Proportion of samples with % GCV ≤ 25%^a^	≥ 95.7%	100%	≥ 90.9%
Analyst ruggedness^b^, fold	0.93–1.14	0.89–1.18	0.93–1.10
Plate lot ruggedness^b^, fold	0.97–1.01	0.97–1.02	0.93–0.98
Intermediate assay precision^c^, % GCV	15.1%	10.2%	14.9%
Statistically significant fold increase^d^	1.82	1.51	1.81
Relative accuracy range	112–117%	107–113%	113–118%
Dilutional linearity^e^, fold	1.11	1.16	1.07
Selectivity^f^	102%	104%	103%
Homologous specificity^g^	> 91%	> 94%	> 95%
Heterologous seasonal coronavirus antigen (OC43) and H3 influenza antigen specificity^g^	< 11%	< 5%	≤ 6%

AU, arbitrary units; ECL, electrochemiluminescence; GCV, geometric coefficient of variation; H3, Hong Kong hemagglutinin subtype 3; LLOQ, lower limit of quantitation; LOD, limit of detection; N, nucleocapsid protein; OC43, seasonal coronavirus OC43 spike protein; RBD, receptor‐binding domain; S, spike protein; SARS‐CoV‐2, severe acute respiratory syndrome coronavirus 2; ULOQ, upper limit of quantitation.

^a^ Minimum % across all dilutions.

^b^ Range of overall fold difference.

^c^ Maximum percent GCV across dilutions.

^d^ Calculated as $e^{3\times \sqrt{2\times \sum \sigma^{2}}}$.

^e^ Bias per 10‐fold increase in dilution.

^f^ Overall % recovery.

^g^ Overall % inhibition.

#### Analytical sensitivity and quantifiable range

The limit of detection (LOD) for each antigen was defined as the lowest antibody concentration where the associated assay signal was statistically higher, with > 95% probability, than a blank sample containing no antibody. This assessment was performed on samples tested at the 500‐fold dilution. The LODs for SARS‐CoV‐2 S, RBD and N antigens were determined to be 7, 13 and 7 AU mL^−1^, respectively (Table 1). The limits of quantitation (LOQs) were based on acceptable assay performance with regard to precision and accuracy for which values were within concentrations corresponding to the second lowest and second highest standard curve points, but not less than the LOD for each antigen. The lower LOQ (LLOQ) for SARS‐CoV‐2 S, RBD and N antigens at 1:500‐fold dilution were established to be 33, 204 and 14 AU mL^−1^, respectively. The upper LOQ (ULOQ) for all three SARS‐CoV‐2 antigens at the 1:50 000 dilution was 2 000 000 AU mL^−1^ (Table 1). Therefore, the combination of 500‐fold and 50 000‐fold dilutions selected for these studies provided an assay range of 4–5 logs for all antigens evaluated (33–2 000 000 AU mL^−1^ for S, 204–2 000 000 for RBD and 14–2 000 000 AU mL^−1^ for N).

#### Analytical specificity

**Figure 2:**
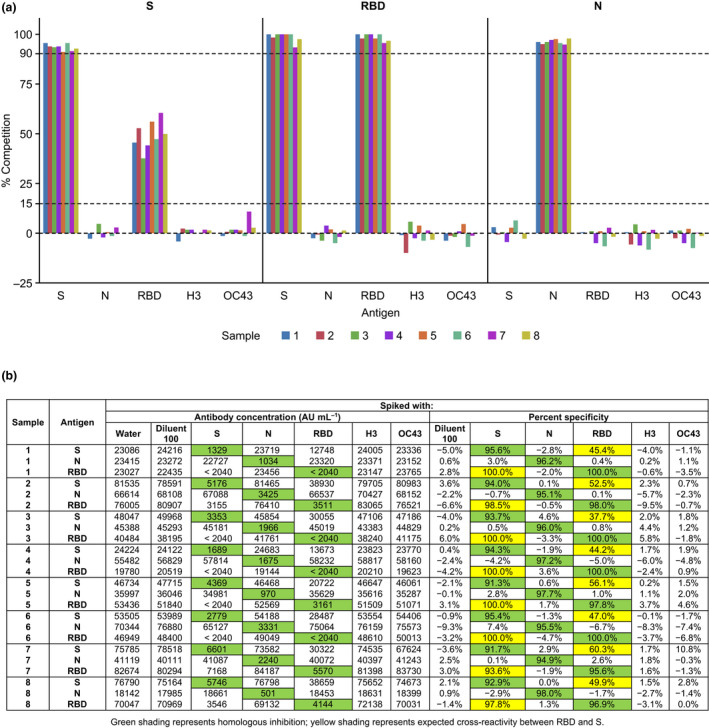
Analytical specificity **(a)** and sensitivity **(b)** of the multiplex SARS‐CoV‐2 ECL serology assay to measure antibodies to heterologous and homologous antigens. ECL, electrochemiluminescence; H3, H3 Hong Kong influenza hemagglutinin; N, nucleocapsid protein; OC43, seasonal coronavirus OC43 spike protein; RBD, receptor‐binding domain; S, spike protein; SARS‐CoV‐2, severe acute respiratory syndrome coronavirus 2.

Competition with homologous and heterologous antigens was performed to determine the analytical specificity of the assay to measure SARS‐CoV‐2‐specific antibodies. Serum samples (*n* = 8) spiked with homologous antigens (SARS‐CoV‐2 S, RBD and N) exhibited > 90% reduction in antibody levels, whereas < 15% reduction in antibody levels was observed when competed with heterologous antigens from other viruses, including the seasonal coronavirus OC43 S and influenza H3 Hong Kong hemagglutinin (Table 1). In addition, when S was used for competition, > 90% reduction in antibody levels was observed for both the full‐length S and RBD antigens. Similarly, when samples were spiked with RBD, > 90% reduction in antibody levels was observed for the RBD antigen, but only a partial reduction in antibody levels (> 37%) were observed for the S antigen. Since the RBD comprises part of the S, it was expected that some of the antibodies that bind to the S antigen would also bind to the RBD moiety (Figure 2a and b).

#### Serostatus cut points

**Figure 3:**
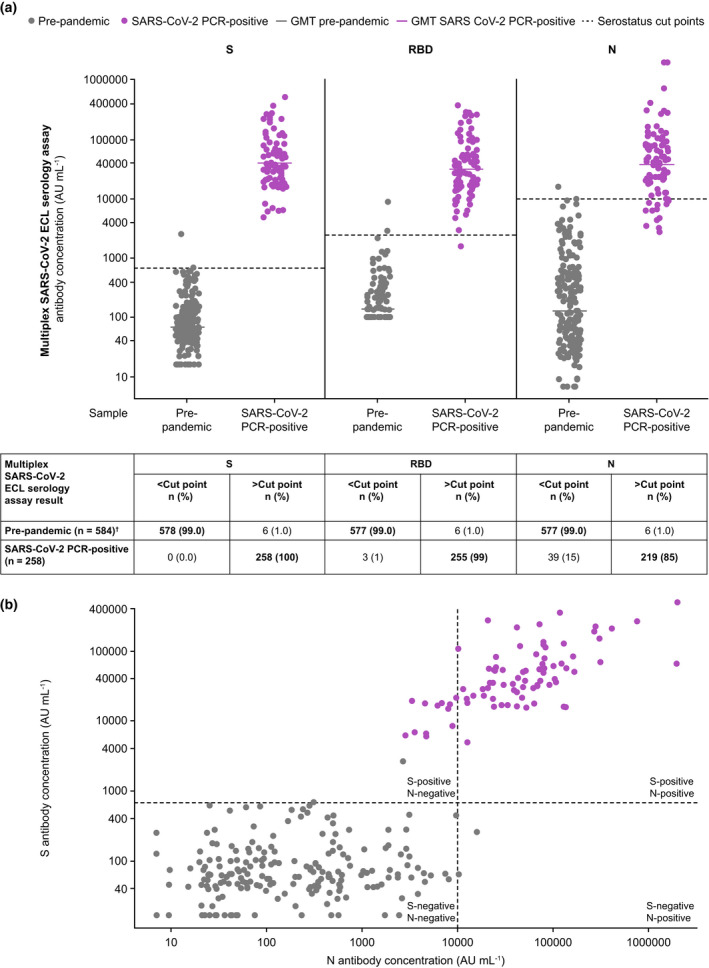
SARS‐CoV‐2 S, RBD and N antibody distribution by serostatus cut points (dashed lines) in samples from donors according to known SARS‐CoV‐2 status **(a)** and number of PCR‐positive samples that were seronegative versus seropositive for both S and N **(b)** ^†^One measurement failed run validity criteria. In **a**, GMT values for each group are shown as solid‐coloured lines. In **b**, RBD versus N distribution is the same as S versus N and is not shown. AU, arbitrary units; ECL, electrochemiluminescence; GMT, geometric mean titre; N, nucleocapsid protein; PCR, polymerase chain reaction; RBD, receptor‐binding domain; S, spike protein; SARS‐CoV‐2, severe acute respiratory syndrome coronavirus 2.

To determine whether an individual had been previously exposed to the SARS‐CoV‐2 virus, we determined the serostatus cut points for the three antigens to differentiate a seropositive versus a seronegative individual. To determine the baseline for a seronegative status, a set of prepandemic samples (*n* = 195) were tested and used to establish the 99th percentile serostatus cut points for the multiplex SARS‐CoV‐2 ECL serology assay. The assay cut points for S, RBD and N SARS‐CoV‐2 antigens were set at 675, 2396 and 9787 AU mL^−1^, respectively (Figure 3a, Table 2). S, RBD and N antibody concentrations were subsequently compared for all serum samples (258 measurements from 86 samples from SARS‐CoV‐2 PCR‐positive individuals and 584 measurements from 195 SARS‐CoV‐2‐negative, prepandemic samples). The assay cut points were applied to the data to determine the agreement between serostatus and SARS‐CoV‐2 prior infection status by PCR (Figure 3b, Table 2) and to calculate clinical sensitivity and specificity of the multiplex SARS‐CoV‐2 ECL serology assay, as described below.

**Table 2:** Multiplex SARS‐CoV‐2 ECL serology assay clinical sensitivity and specificity

Sensitivity analyses from SARS‐CoV‐2–PCR‐positive individuals^a^				
SARS‐CoV‐2 antigen	Serostatus cut point, AU mL^−1^	Measurements, *n*	Samples ≥ cut point, *n*	Sensitivity, % (95% CI)
S	675	258	258	100 (98.6, 100)
RBD	2396	258	255	98.8 (96.6, 99.8)
N	9787	258	219	84.9 (79.9, 89.0)

Specificity analyses from SARS‐CoV‐2‐negative individuals^b^				
SARS‐CoV‐2 antigen	Serostatus cut point, AU mL^−1^	Measurements, *n*	Samples < cut point, *n*	Specificity, % (95% CI)
S	675	584	578	99.0 (97.8, 99.6)
RBD	2396	583	577	99.0 (97.8, 99.6)
N	9787	583	577	99.0 (97.8, 99.6)

AU, arbitrary units; CI, confidence interval; ECL, electrochemiluminescence; N, nucleocapsid protein; PCR, polymerase chain reaction; RBD, receptor‐binding domain; S, spike protein; SARS‐CoV‐2, severe acute respiratory syndrome coronavirus 2.

^a^ Samples for the sensitivity analyses were collected ≥ 14 days after obtaining a positive PCR test result.

^b^ Samples for the specificity analyses were obtained prepandemic.

#### Clinical sensitivity and specificity

A total of 258 measurements from 86 serum samples from SARS‐CoV‐2 PCR‐positive individuals (collected ≥ 14 days after obtaining a positive PCR result) were used to assess clinical sensitivity or the ability to correctly identify the samples from individuals previously diagnosed with SARS‐CoV‐2 infection (true positive rate). This assessment was performed for each of the three SARS‐CoV‐2 antigens, and the clinical sensitivities were determined as the proportion of samples at or above the cut point. For SARS‐CoV‐2 S, RBD and N antigens, the clinical sensitivity of each serology assay was 100% (258/258), 98.8% (255/258) and 84.9% (219/258), respectively (Table 2).

Conversely, a total of 584 measurements from 195 SARS‐CoV‐2‐negative, prepandemic samples were used to assess clinical specificity, or the ability to correctly identify those individuals without prior SARS‐CoV‐2 infection (true negative rate), as determined by the number of measurements below the cut point for SARS‐CoV‐2 S, RBD and N antigens. For all three antigens, clinical specificity was 99.0% (Table 2).

### Concordance with FDA‐authorised Elecsys® assay and a SARS‐CoV‐2 neutralisation assay

**Figure 4:**
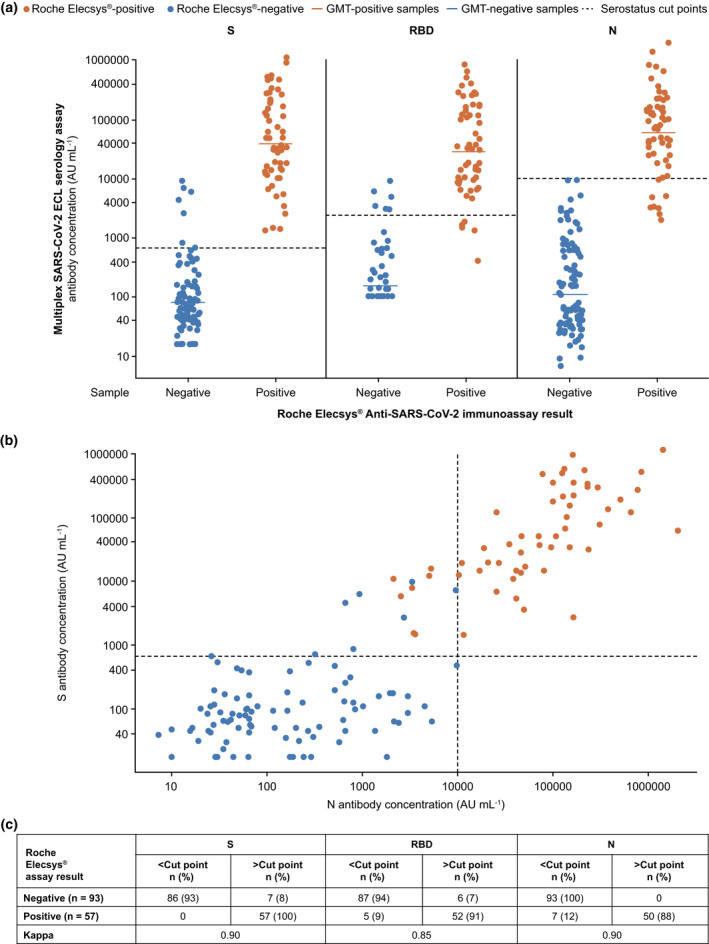
SARS‐CoV‐2 S, RBD and N antibody distribution by serostatus cut points (dashed lines) in samples from donors with known Roche Elecsys® Anti‐SARS‐CoV‐2 immunoassay results **(a)** and distribution of S versus N antibody concentrations as measured by the multiplex SARS‐CoV‐2 ECL serology assay **(b)**. In **a,** GMT values for each group are shown as solid‐coloured lines. In **b,** RBD versus N distribution is the same as S versus N and is not shown. AU, arbitrary units; ECL, electrochemiluminescence; GMT, geometric mean titre; N, nucleocapsid protein; RBD, receptor‐binding domain; S, spike protein; SARS‐CoV‐2, severe acute respiratory syndrome coronavirus 2.

To evaluate the performance of the multiplex SARS‐CoV‐2 ECL serology assay with an FDA‐authorised sero‐diagnostic assay, we compared the serostatus results to the Roche Elecsys® Anti‐SARS‐CoV‐2 immunoassay, a diagnostic assay for detecting antibodies to the SARS‐CoV‐2 N antigen. The 99^th^ percentile serostatus cut points for each of the SARS‐CoV‐2 antigens were additionally applied to the antibody concentration of 150 samples with known Roche Elecsys® Anti‐SARS‐CoV‐2 immunoassay results (Figure 4a). Distribution of S versus N antibody concentrations as measured by the multiplex SARS‐CoV‐2 ECL serology assay is shown in Figure 4b.

A separate supplementary sample set (*n* = 150), different from the sample set used to determine the serostatus cut points, and with unknown SARS‐CoV‐2 PCR status, was also tested in both assays. For the 57 samples that were detected as seropositive with the Roche Elecsys® Anti‐SARS‐CoV‐2 immunoassay, ≥ 87.7% of them were above all antigen cut points (i.e. positive) in the multiplex SARS‐CoV‐2 ECL serology assay, with 100% agreement for the S antigen (Figure 4c). For the remaining 93 samples that were classified as seronegative by the Roche Elecsys® Anti‐SARS‐CoV‐2 immunoassay, ≥ 92% were below all antigen cut points (i.e. negative) in the multiplex SARS‐CoV‐2 ECL serology assay, with 100% agreement for the N antigen (Figure 4c). For S, RBD and N assays, kappa scores were determined to be 0.90, 0.85 and 0.90, respectively, demonstrating a strong level of agreement between the multiplex SARS‐CoV‐2 ECL serology assay and the Roche Elecsys® Anti‐SARS‐CoV‐2 immunoassay (Figure 4c).

**Figure 5:**
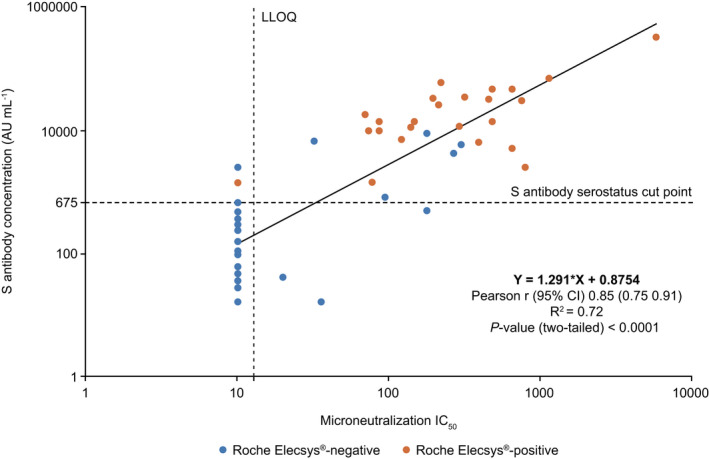
Correlation between the multiplex SARS‐CoV‐2 ECL serology assay (S assay) and the BBRC SARS‐CoV‐2 live‐virus MN assay. A total of 57 samples were tested in three assays. Horizontal dotted line indicates the multiplex SARS‐CoV‐2 ECL serology S assay cut point (675 AU mL^−1^). Vertical dotted line indicates BBRC MN assay LLOQ (IC_50_ = 20). Colour shows Roche Elecsys® Anti‐SARS‐CoV‐2 immunoassay results. AU, arbitrary units; BBRC, Battelle Biomedical Research Center; CI, confidence interval; ECL, electrochemiluminescence; IC_50_, half maximal inhibitory concentration; LLOQ, lower limit of quantitation; MN, microneutralisation; S, spike protein; SARS‐CoV‐2, severe acute respiratory syndrome coronavirus 2.

The quantitative values generated in the multiplex SARS‐CoV‐2 ECL serology assay were compared with neutralisation antibody titres determined in the BBRC SARS‐CoV‐2 live‐virus MN assay, a cell‐based assay that measures the ability of antibodies to neutralise replication‐competent SARS‐CoV‐2. Comparison of the antibody titres and concentrations of 57 samples demonstrated concordance (Pearson *r* = 0.85, *R* ^2^ = 0.72, *P < 0.0001) between the multiplex SARS‐CoV‐2 ECL serology assay and the BBRC SARS‐CoV‐2 live‐virus MN assay. These 57 samples were a subset of 150 samples tested in the Roche Elecsys® Anti‐SARS‐CoV‐2 immunoassay as previously discussed. All 24 samples that were detected as seropositive in the Roche Elecsys® Anti‐SARS‐CoV‐2 immunoassay were above the S antigen cut point for the multiplex SARS‐CoV‐2 ECL serology assay, and 23 of 24 samples were positive in the BBRC SARS‐CoV‐2 live‐virus MN assay (Figure 5). Of the 33 samples that were detected as seronegative in the Roche Elecsys® Anti‐SARS‐CoV‐2 immunoassay, six were above the S antigen cut point in the multiplex SARS‐CoV‐2 ECL serology assay, and five of these showed neutralisation activity in the BBRC SARS‐CoV‐2 live‐virus MN assay (Figure 5).

### Assay calibration to the WHO international and MSD reference standards

#### WHO international standard NIBSC 20/136

A series of experiments were performed to calibrate the AstraZeneca (AZ) reference standard used in the validation experiments performed at PPD® Laboratories (Richmond, VA, USA) to the WHO International Standard 20/136 (National Institute for Biological Standards and Controls [NIBSC], Potters Bar, Hertfordshire, UK).^16^ It was determined that a serum sample with an IgG concentration in AU mL^−1^ calculated from the AZ reference standard in the multiplex SARS‐CoV‐2 ECL serology assay can be converted to WHO binding antibody units (BAU) mL^−1^ by multiplying the concentration by the appropriate conversion factor. The WHO International Standard conversion factors established were 0.00645, 0.00798 and 0.00324 for the SARS‐CoV‐2 S, RBD and N antigens, respectively (Table 3).

**Table 3:** Conversion factors calibrating the multiplex SARS‐CoV‐2 ECL serology assay arbitrary units (AU mL^−1^) to the WHO International Reference Standard units (BAU mL^−1^)

SARS‐CoV‐2 antigen	WHO International Standard	
	Conversion factor (AU mL^−1^ to BAU mL^−1^)	95% CI
S	0.00645	0.00594–0.00701
RBD	0.00798	0.00735–0.00866
N	0.00324	0.00295–0.00356

AU, arbitrary units; BAU, binding arbitrary units; CI, confidence interval; ECL, electrochemiluminescence; N, nucleocapsid protein; RBD, receptor‐binding domain; S, spike protein; SARS‐CoV‐2, severe acute respiratory syndrome coronavirus 2; WHO, World Health Organization.

#### MSD reference standard 1

A series of experiments were performed to calibrate the AZ reference standard to the now commercially available and widely distributed MSD Reference Standard 1 (see Methods for further details), which was not available when the validation experiments described in this manuscript were performed. The conversion factors for the AZ reference standard (AU mL^−1^) to the MSD Reference Standard 1 (MSD AU mL^−1^) were calculated to be 0.82236, 0.30834 and 2.03375 for the SARS‐CoV‐2 S, RBD and N antigens, respectively.

## Discussion

In this study, we describe the validation of a quantitative, multiplexed, high‐throughput, sensitive, specific, SARS‐CoV‐2 IgG serology assay for fast and accurate detection of SARS‐CoV‐2 antibodies in human sera. This assay has been used to measure antibodies to SARS‐CoV‐2 S, RBD and N antigens simultaneously, in support of multiple phase three clinical trials of a SARS‐CoV‐2 vaccine^17^, ^18^ (with > 50 000 clinical trial biospecimens tested to date). This assay may additionally enable large‐scale sero‐epidemiology studies to determine the prevalence or incidence of SARS‐CoV‐2 infections and support immunisation programs around the world.

Although serology assays differ from typical immunoassays, the standard validation parameters (sensitivity, specificity, precision, linearity and accuracy) of the multiplex SARS‐CoV‐2 ECL serology assay were characterised, and all met the acceptance criteria in the validation plan. Notably, the assay demonstrated a broad quantifiable range, which allows most samples to be tested at a single dilution, significantly increasing the throughput of testing clinical samples. The assay also demonstrated acceptable ruggedness, precision, relative accuracy, dilutional linearity (< 2‐fold per 10‐fold increase in dilution), and clinical sensitivity and specificity, as well as analytical specificity. With respect to the latter, all evaluable samples showed > 90% reduction in antibody levels when spiked with homologous antigens, and < 15% reduction in antibody levels when spiked with heterologous antigens, including heterologous seasonal coronavirus OC43 spike and H3 Hong Kong influenza hemagglutinin antigens. Expected cross‐reactivity of serum antibodies between SARS‐CoV‐2 RBD and S antigens was demonstrated, given that the S contains the RBD as one of its two protein subunits.^5^

Classification of samples as seronegative or seropositive based on the serostatus cut points for the multiplex SARS‐CoV‐2 ECL serology assay demonstrated good agreement (≥ 85%) with the previously established SARS‐CoV‐2 status of the samples based on a documented SARS‐CoV‐2 infection by PCR. We note that our assay serostatus cut points were developed utilising pre‐pandemic samples obtained from male donors. As gender has been associated with modest impacts on immunogenicity from vaccination and viral pathogenesis, we acknowledge this as a potential limitation of our analysis.^19^, ^20^ However, our cut points were tested using confirmed SARS‐CoV‐2 PCR‐positive samples obtained from male and female donors. Furthermore, the assay demonstrated concordance with the FDA‐authorised Roche Elecsys® Anti‐SARS‐CoV‐2 immunoassay, which is used as a diagnostic assay for detecting antibodies to the SARS‐CoV‐2 N antigen.^11^ The multiplex SARS‐CoV‐2 ECL serology assay also demonstrated concordance with the BBRC SARS‐CoV‐2 live‐virus MN assay. Live‐virus neutralisation assays are considered the ‘gold standard’ for measuring neutralising antibody levels, but are laborious, in the case of SARS‐CoV‐2 require biosafety level three facilities,^21^, ^22^ and are subject to assay variation because of different virus lots, cell lots and other key reagents.^23^

The multiplex SARS‐CoV‐2 ECL serology assay was also calibrated to the WHO International Standard (NIBSC 20/136) and the commercially available MSD Reference Standard 1. Calibration to both standards will assist evaluation of vaccine immunogenicity studies and aid comparison of data collected from multiple vaccine manufacturers as part of epidemiological and immunological surveillance studies.^8^, ^22^

There is broad applicability of a reproducible SARS‐CoV‐2 serological assay to support COVID‐19 vaccine and mAb clinical studies. In particular, measuring antibody levels over time will help determine whether there is a minimum protective level of antibodies associated with protection and help determine the duration of protection afforded by vaccination or administration of mAbs.^22^, ^24^ Furthermore, a multiplex assay that can identify epitope‐specific responses following vaccination or infection could provide insight into correlates of protection,^25^ and high‐quality antibody tests can help broaden the understanding of the humoral antibody response.^26^ SARS‐CoV‐2 infections are diagnosed predominantly with molecular testing of respiratory samples, which allows early detection of infection but cannot be used to determine the overall exposure in a community to support epidemiology studies or gauge the level of herd immunity.^27^ Rapid molecular tests are costly with low throughput, while high‐throughput tests have longer turnaround times owing to the time requirements of sample extraction.^28^, ^29^ To mitigate these limitations, antibody tests could be incorporated into the diagnostic arsenal for COVID‐19.^22^, ^27^ Easy to perform, high‐throughput serology assays could also improve understanding of the impact of the SARS‐CoV‐2 virus through large sero‐epidemiologic studies, which is particularly helpful given that as many as 45% of SARS‐CoV‐2 infections may be asymptomatic^30^ and those with mild COVID‐19 symptoms are mostly undetected^31^ and may prove even more valuable when correlates of protection of vaccines are established and recognised.^32^

Having the capability to quantify antibody levels to SARS‐CoV‐2 would inform the evolving scientific understanding of viral prevalence, transmission dynamics, disease burden and rates of symptomatic and asymptomatic infection, all of which impact the public health response to this virus.^22^ Because the multiplex SARS‐CoV‐2 ECL serology assay measures antibodies to both S and N, it has the ability to distinguish the previously infected/convalescent individuals with antibodies to S, RBD and N antigens from vaccinated individuals who would only have S and RBD antibodies, but not N antibodies. Our investigation complements similar findings by Hicks *et al.,* ^33^ who developed an enzyme‐linked immunosorbent assay (ELISA)‐based assay to determine the seroprevalence of SARS‐CoV‐2 in the Australian population. In order to compensate for poor S1 antibody sensitivity and specificity, Hicks *et al*. employed an alternative methodology by which the mean concentrations of N and RBD antibodies were combined to increase overall assay sensitivity. However, we believe our approach, of keeping the assay cut points separate, is more suitable for characterising the vaccine‐induced immune response, for which antigen specificity between N, S and RBD is the most important characteristic. We show N and S antibody levels for each donor within our analysis.

A major limitation of traditional serological assay techniques, such as ELISA, is their narrow dynamic range and low throughput, which do not allow for unequivocal and unambiguous interpretation of results.^34^ This would be a particular issue for SARS‐CoV‐2 detection because of potential for significant cross‐reactivity to other seasonal coronavirus infections.^22^, ^35^ Specificity can be augmented by evaluating humoral immunity to more than one viral‐associated antigen at a time by using multiplexing.^36^ In contrast to an ELISA, the multiplex SARS‐CoV‐2 ECL serology assay described herein can measure several SARS‐CoV‐2 antigen‐specific antibodies simultaneously, without requirements for additional sample volume, with added advantages of a large dynamic range, sensitivity and ruggedness of performance.

Validated antibody assays that are sensitive, precise, accurate and reproducible under a variety of test conditions enable robust testing of serum samples from vaccine and therapeutic antibody clinical trials. Validated assays also provide data that offer insights into the kinetics of antibody responses to SARS‐CoV‐2 following infection or vaccination and the potential duration of protection. Antibody levels from infections in convalescent individuals or breakthrough cases in immunised individuals in clinical trials can help determine a correlate of protection. All the assay parameters met the acceptance criteria, and the assay reference standard (AZ reference standard) has been calibrated to the WHO International and MSD reference standards. The multiplex SARS‐CoV‐2 ECL serology assay is considered validated and fit for its intended purpose of measuring SARS‐CoV‐2‐specific IgG antibodies in registrational clinical trials and sero‐epidemiology studies.

## Methods

### Multiplex SARS‐CoV‐2 ECL serology assay components

All experiments utilised Meso Scale Discovery (Rockville, MD, USA; MSD) custom SARS‐CoV‐2 MULTI‐SPOT® plates (Lots Z0056737 and Z0056738), which were directly coated with antigen proteins, including S, RBD and N of SARS‐CoV‐2. Additional components of the assay included MSD® Diluent 100 (Catalogue # R50AA), MSD® Blocker A Solution (Catalogue # R93BA), MSD® GOLD™ Read Buffer B (Catalogue # R60AM), MSD® SULFO‐TAG™‐labelled IgG detection antibody (mouse anti‐human IgG; Lot # D00V0003) and MSD® Wash Buffer (20X; Catalogue # R61AA). All components listed above are now part of the MSD V‐PLEX® SARS‐CoV‐2 Panel 2 (IgG) Kit (Catalog # K15383U). At the time of validation, the kit was not available, and MSD provided custom components for the work. Prior to the work described here, assay components including antigens, reference standard, plates, diluents and detection antibody were characterised, and assay optimisation was performed by MSD. Additionally, because the MSD Reference Standard 1 was not available, the AZ reference standard (not provided by MSD) was created (see below).

### Serum samples for reference serum, controls and validation panel

The AstraZeneca reference standard was made by pooling sera from 10 convalescent donors containing antibodies specific for the SARS‐CoV‐2 S, RBD and N antigens. Four different quality control samples (QCS) were made by pooling sera from 4 or 5 convalescent donors and selected to be at approximately 20%, 50% and 80% of the maximum assay signal. Additionally, a set of individual convalescent serum samples was used during validation. Sera used for specificity analyses pre‐dated November 2019 and were deemed prepandemic. These prepandemic serum samples (*n* = 195) were derived from anonymised male donors aged 18–80 years (with a median age of 40.5 years) and were prescreened to have low or negative SARS‐CoV‐2 antibody concentrations as measured in the multiplex ECL serology assay. All serum samples were obtained from a commercial vendor (BioIVT, Westbury, NY, USA).

### Serum samples for cut point analysis

Serum samples confirmed as SARS‐CoV‐2 PCR‐positive were obtained from a commercial vendor (BioIVT). These confirmed SARS‐CoV‐2 PCR‐positive serum samples (*n* = 86) were obtained from 46 female and 40 male donors aged 18–82 years (with a median age of 40.5 years). Serum samples were collected 14–30 days after a SARS‐CoV‐2‐positive PCR result for 29 donors and 30–60 days after a SARS‐CoV‐2‐positive PCR result for 57 donors. SARS‐CoV‐2 prepandemic samples were obtained from a commercial vendor (BioIVT) and were collected prior to November 2019.

### Assay protocol

A MULTI‐SPOT® 96‐well plate was coated with SARS‐CoV‐2 S, RBD and N antigens (MSD® SARS‐CoV‐2 Plate 2). To measure IgG antibodies to SARS‐CoV‐2 antigens, plates were blocked with MSD Blocker A for 1 h and washed prior to the addition of reference standard, controls and samples. After incubation for 2 h, the plates were washed and detection antibody was added (MSD® SULFO‐TAG™ Anti‐Human IgG Antibody). Plates were incubated for 1 h and washed three times. MSD GOLD™ Read Buffer B was added, and the plates were read using a MESO® SECTOR S 600 Reader. The AstraZeneca reference standard and four QCS, as previously described, were included on each plate in routine operation of the assay, and the plate was repeated if two or more QCS exceeded their respective 2σ limits.

### Assay validation and characterisation

Assay validation and characterisation were conducted by PPD® Laboratories (Richmond, VA, USA). A series of studies, as described below, were performed to determine the precision, linearity, ruggedness, sensitivity and specificity of the multiplex SARS‐CoV‐2 ECL serology assay in detecting three SARS‐CoV‐2 antigen‐specific antibodies (S, RBD and N) in serum samples.

#### Standard curve characterisation

Standard curve modelling was performed using an 11‐point and 2.5‐fold dilution series of the reference standard on each assay plate. The associated standard curve starting concentration was arbitrarily assigned to 100 000 AU mL^−1^ for each antigen, and a 4‐parameter logistic function was used to model the 2.5‐fold standard dilution series for each plate. ECL signals were quantified in relative light units (RLU). Based on RLU values and variability estimates for each standard curve point, RLU values < 200 were not included in the fit for the standard curve because of high variability. The standard curve criteria permitted dropping ≤ 2 points with RLU values < 200 for an appropriate and adequate fit to the model.

#### Assay precision, dilutional linearity and ruggedness

Assay precision, dilutional linearity and ruggedness were assessed by using 21 samples prescreened to have antibody concentrations that span the quantifiable range analysed across 15 runs by five analysts utilising two plate lots at three dilutions (1:500, 1:5000 and 1:50 000). Variability estimates were obtained using variance component analysis and expressed as the per cent geometric coefficient of variation (% GCV) calculated as % GCV = 100% ×${[e}^{\sqrt{{\hat{\sigma }}^{2}}\;}‐1]$ for assay precision where ${\hat{\sigma }}^{2}$ represents the variance of the natural log‐ (*ln*) transformed antibody concentrations. Intra‐assay precision was evaluated using four quality controls on each plate. For dilutional linearity, dilution bias per 10‐fold dilution was estimated across all dilutions tested, and the assay was considered to be dilutable if the dilution bias per 10‐fold dilution was < 2‐fold difference. Assay ruggedness was assessed by comparing fold differences in antibody concentrations between analysts and plate lots and considered to be acceptable if within ± 1.3‐fold for each antigen tested. Meaningful fold‐rise was calculated as $e^{3\times \sqrt{2\times \sum {\hat{\sigma }}^{2}}}$, where ${\hat{\sigma }}^{2}$ represents the variance of the natural log‐ (*ln*) transformed antibody concentrations.

#### Analytical sensitivity and quantifiable range

The LOD was determined using mock samples prepared with increasing antibody concentrations. Differences in RLU values between the mock samples and the blank samples containing no added antibody were compared. The differences were determined separately within each plate, run in combination and then averaged across plates/runs for each spike level. The standard deviations of the differences at each antibody level were calculated. The LOD was set at the antibody level that provided a statistically significant increase in RLU above that in the blank sample, where significance was based on a *t*‐distribution at the 5% significance level and determined using the mean and standard deviation of the individual differences.

The LLOQ and ULOQ for a single dilution for each of the three SARS‐CoV‐2 antigen‐specific antibodies were determined by evaluating the precision profiles of the antibody concentrations and the relative accuracy of the assay. The dynamic range of the assay was evaluated by testing spiked serum samples at three different dilutions for each antigen, S, RBD and N.

#### Analytical specificity

Analytical specificity of the assay was evaluated using SARS‐CoV‐2 S, RBD and N antigens for homologous competition, and seasonal coronavirus OC43 S and H3 Hong Kong influenza hemagglutinin antigens for heterologous competition. Competition experiments were performed by spiking homologous or heterologous antigens into eight human serum samples with mid‐to‐high range antibody concentrations. Assay specificity was determined by comparing antibody concentration of serum spiked with antigens to the antibody concentration of the serum samples that were not spiked with antigens.

During development of the assay, MSD performed competition experiments with additional heterologous antigens, including SARS CoV‐1, Middle East respiratory syndrome coronavirus (MERS‐CoV) and human coronavirus HKU1 (hCOV‐HKU1) spike proteins. Although this analysis was not completed using the final method, observed cross‐reactivity to these heterologous antigens was comparable to OC43 S (signals within two‐fold of ‘unspiked’ serum), confirming the assays are specific (data not shown).

#### Serostatus cut points

The antigen cut point values were established by running 585 measurements from 195 prepandemic serum samples run in triplicate in the assay. Cut points for each antigen were determined using the 99th percentile on the natural log‐transformed sample antibody concentrations.

#### Clinical sensitivity and specificity

The 99th percentile cut point based on the SARS‐CoV‐2 known negative prepandemic samples was used to calculate clinical sensitivity and specificity.

### Concordance with FDA‐authorised Elecsys® assay and a SARS‐CoV‐2 neutralisation assay

Serostatus cut points established for the multiplex SARS‐CoV‐2 ECL serology assay were applied to a subset of 150 samples with known results using the Roche Elecsys® Anti‐SARS‐CoV‐2 immunoassay (i.e. a diagnostic assay for detecting antibodies to the SARS‐CoV‐2 N antigen). This subset of samples was also assessed for neutralisation activity using the BBRC SARS‐CoV‐2 live‐virus MN assay. The Roche Elecsys® Anti‐SARS‐CoV‐2 immunoassay is an ECL immunoassay that uses a recombinant protein representing nucleocapsid (N) antigen for determination of antibodies against SARS‐CoV‐2. The Roche Elecsys® Anti‐SARS‐CoV‐2 immunoassay was performed by PPD® Laboratories (Highland Heights, KY, USA). The BBRC SARS‐CoV‐2 live‐virus MN assay was developed, validated and performed at BBRC (Columbus, OH, USA). The BBRC assay is a cell‐based, wild‐type live‐virus MN assay with an *in situ* ELISA readout that detects SARS‐CoV‐2 viral protein in a fixed VERO E6 monolayer. The reciprocal of the highest dilution of test sample with an optical density less than the neutralising plate cut off (50% of the viral signal) is reported as the endpoint titre. The half‐maximal inhibitory concentration (IC_50_) is determined by logistic regression analysis. Assay concordance was determined by assessing percentage agreement between the positive and negative samples between the multiplex SARS‐CoV‐2 ECL serology assay and the Roche Elecsys® Anti‐SARS‐CoV‐2 immunoassay and also by calculating Pearson correlation between the multiplex SARS‐CoV‐2 ECL serology S assay (AU mL^−1^) and BBRC MN assay (IC_50_). Assay results were log‐transformed prior to calculating Pearson correlation.

### Assay calibration to the WHO international and MSD reference standards

#### WHO international reference standard (NIBSC 20/136)

The First WHO International Standard of anti‐SARS‐CoV‐2 IgG NIBSC 20/136 Version 2.0 was used in the study.^16^ It consists of pooled plasma samples obtained from individuals recovered from SARS‐CoV‐2 infection and was evaluated in a WHO international collaborative study.^37^ The intended use of the WHO International Standard is for the calibration and harmonisation of serological assays detecting anti‐SARS‐CoV‐2 neutralising antibodies. Per product instructions, the assigned potency of the WHO International Standard for SARS‐CoV‐2 (NIBSC 20/136) for binding antibody assays is an arbitrary unitage of 1000 BAU mL^−1^ and can be used to assist in the comparison of assays detecting the same class of IgGs with the same specificity (e.g. anti‐RBD IgG or anti‐N IgG). To anchor the multiplex SARS‐CoV‐2 ECL serology assay unitage to the WHO International Standard NIBSC 20/136, a series of calibration experiments were performed to interpolate the WHO International Standard RLUs from the AZ reference standard for the SARS‐CoV‐2 S, RBD and N antigens to derive conversion factors for AU mL^−1^ to BAU mL^−1^.

#### MSD reference standard 1

A series of calibration experiments were also performed to interpolate the MSD Reference Standard 1 (component of the V‐PLEX® SARS‐CoV‐2 Panel 2 Kit, Catalog Number K15383U, Lot A00V0004) RLUs to the AZ reference standard used in the development and validation of the multiplex SARS‐CoV‐2 ECL serology assay and to derive conversion factors for AZ AU mL^−1^ to MSD AU mL^−1^.

## Conflict of Interest

DW, AAA, AR, KMT, MTE and EJK are employees of AstraZeneca and may hold stock or stock options. TG, RG, BF and CJBare employees of PPD® Laboratories and may hold stock or stock options.

## Author Contributions

**Deidre Wilkins:** Conceptualization; Formal analysis; Methodology; Supervision; Validation; Writing – original draft; Writing – review & editing. **Anastasia A Aksyuk:** Conceptualization; Formal analysis; Methodology; Validation; Writing – original draft; Writing – review & editing. **Alexey Ruzin:** Conceptualization; Formal analysis; Writing – original draft; Writing – review & editing. **Kevin M Tuffy:** Formal analysis; Writing – review & editing. **Tina Green:** Formal analysis; Validation; Writing – review & editing. **Rebecca Greway:** Validation; Writing – review & editing. **Brittany Fikes:** Data curation; Methodology; Validation; Writing – review & editing. **Cyrille J Bonhomme:** Data curation; Methodology; Validation; Writing – review & editing. **Mark T Esser:** Conceptualization; Supervision; Writing – review & editing. **Elizabeth J Kelly:** Conceptualization; Supervision; Validation; Writing – review & editing.

## References

[1] Chan JF , Kok KH , Zhu Z *et al*. Genomic characterization of the 2019 novel human‐pathogenic coronavirus isolated from a patient with atypical pneumonia after visiting Wuhan. Emerg Microbes Infect 2020; 9: 221–236. 31987001 10.1080/22221751.2020.1719902 PMC7067204

[2] Zhou P , Yang XL , Wang XG *et al*. A pneumonia outbreak associated with a new coronavirus of probable bat origin. Nature 2020; 579: 270–273. 32015507 10.1038/s41586-020-2012-7 PMC7095418

[3] Phan T . Novel coronavirus: from discovery to clinical diagnostics. Infect Genet Evol 2020; 79: 104211. 32007627 10.1016/j.meegid.2020.104211 PMC7129799

[4] Cheng MP , Papenburg J , Desjardins M *et al*. Diagnostic testing for severe acute respiratory syndrome‐related coronavirus 2: a narrative review. Ann Intern Med 2020; 172: 726–734. 32282894 10.7326/M20-1301 PMC7170415

[5] Huang Y , Yang C , Xu XF , Xu W , Liu SW . Structural and functional properties of SARS‐CoV‐2 spike protein: potential antivirus drug development for COVID‐19. Acta Pharmacol Sin 2020; 41: 1141–1149. 32747721 10.1038/s41401-020-0485-4 PMC7396720

[6] World Health Organization . WHO coronavirus (COVID‐19) dashboard. Access date: March 08, 2021. Available from: https://covid19.who.int/

[7] Cucinotta D , Vanelli M . WHO declares COVID‐19 a pandemic. Acta Biomed 2020; 91: 157–160. 32191675 10.23750/abm.v91i1.9397 PMC7569573

[8] Johnson M , Wagstaffe HR , Gilmour KC *et al*. Evaluation of a novel multiplexed assay for determining IgG levels and functional activity to SARS‐CoV‐2. J Clin Virol 2020; 130: 104572. 32769024 10.1016/j.jcv.2020.104572 PMC7396134

[9] Le TT , Cramer JP , Chen R , Mayhew S . Evolution of the COVID‐19 vaccine development landscape. Nat Rev Drug Discov 2020; 19: 667–668. 32887942 10.1038/d41573-020-00151-8

[10] Kallolimath S , Sun L , Palt R *et al*. Highly active engineered IgG3 antibodies against SARS‐CoV‐2. Proc Natl Acad Sci USA 2021; 118: e2107249118. 34599091 10.1073/pnas.2107249118 PMC8545452

[11] Muench P , Jochum S , Wenderoth V *et al*. Development and validation of the elecsys anti‐SARS‐CoV‐2 immunoassay as a highly specific tool for determining past exposure to SARS‐CoV‐2. J Clin Microbiol 2020; 58: e01694–20. 32747400 10.1128/JCM.01694-20 PMC7512151

[12] Marchese RD , Puchalski D , Miller P *et al*. Optimization and validation of a multiplex, electrochemiluminescence‐based detection assay for the quantitation of immunoglobulin G serotype‐specific antipneumococcal antibodies in human serum. Clin Vaccine Immunol 2009; 16: 387–396. 19158284 10.1128/CVI.00415-08 PMC2650878

[13] Gilbert PB , Montefiori DC , McDermott AB *et al*. Immune correlates analysis of the mRNA‐1273 COVID‐19 vaccine efficacy clinical trial. Science 2022; 375: 43–50. 34812653 10.1126/science.abm3425 PMC9017870

[14] Debad JD , Glezer EN , Leland JK , Sigal GB . Clinical and biological applications of ECL. In Bard AJ editor, Electrogenerated chemiluminescence. 1st edn: CRC Press; 2004, pp 43–78.

[15] Goldblatt D , Ashton L , Zhang Y , Antonello J , Marchese RD . Comparison of a new multiplex binding assay versus the enzyme‐linked immunosorbent assay for measurement of serotype‐specific pneumococcal capsular polysaccharide IgG. Clin Vaccine Immunol 2011; 18: 1744–1751. 21813660 10.1128/CVI.05158-11 PMC3187042

[16] NIBSC, WHO First WHO international standard for anti‐SARS‐CoV‐2 (NIBSC 20/136). Access date: March 8, 2022. [updated Version 2.0, December 17, 2020]. Available from: https://www.nibsc.org/documents/ifu/20‐136.pdf

[17] Voysey M , Costa Clemens SA , Madhi SA *et al*. Single‐dose administration and the influence of the timing of the booster dose on immunogenicity and efficacy of ChAdOx1 nCoV‐19 (AZD1222) vaccine: a pooled analysis of four randomised trials. Lancet 2021; 397: 881–891. 33617777 10.1016/S0140-6736(21)00432-3 PMC7894131

[18] Falsey AR , Sobieszczyk ME , Hirsch I *et al*. Phase 3 safety and efficacy of AZD1222 (ChAdOx1 nCoV‐19) Covid‐19 vaccine. N Engl J Med 2021; 385: 2348–2360. 34587382 10.1056/NEJMoa2105290 PMC8522798

[19] Bunders MJ , Altfeld M . Implications of sex differences in immunity for SARS‐CoV‐2 pathogenesis and design of therapeutic interventions. Immunity 2020; 53: 487–495. 32853545 10.1016/j.immuni.2020.08.003 PMC7430299

[20] Fathi A , Addo MM , Dahlke C . Sex differences in immunity: implications for the development of novel vaccines against emerging pathogens. Front Immunol 2020; 11: 601170. 33488596 10.3389/fimmu.2020.601170 PMC7820860

[21] Almahboub SA , Algaissi A , Alfaleh MA , ElAssouli MZ , Hashem AM . Evaluation of neutralizing antibodies against highly pathogenic coronaviruses: a detailed protocol for a rapid evaluation of neutralizing antibodies using vesicular stomatitis virus pseudovirus‐based assay. Front Microbiol 2020; 11: 2020. 33013745 10.3389/fmicb.2020.02020 PMC7498578

[22] Cheng MP , Yansouni CP , Basta NE *et al*. Serodiagnostics for severe acute respiratory syndrome‐related coronavirus 2: a narrative review. Ann Intern Med 2020; 173: 450–460. 32496919 10.7326/M20-2854 PMC7281623

[23] Thompson S , Chesher D . Lot‐to‐lot variation. Clin Biochem Rev 2018; 39: 51–60. 30473592 PMC6223607

[24] Whitman JD , Hiatt J , Mowery CT *et al*. Evaluation of SARS‐CoV‐2 serology assays reveals a range of test performance. Nat Biotechnol 2020; 38: 1174–1183. 32855547 10.1038/s41587-020-0659-0 PMC7740072

[25] Shrock E , Fujimura E , Kula T *et al*. Viral epitope profiling of COVID‐19 patients reveals cross‐reactivity and correlates of severity. Science 2020; 370: eabd4250. 32994364 10.1126/science.abd4250 PMC7857405

[26] Poland GA , Ovsyannikova IG , Kennedy RB . SARS‐CoV‐2 immunity: review and applications to phase 3 vaccine candidates. Lancet 2020; 396: 1595–1606. 33065034 10.1016/S0140-6736(20)32137-1 PMC7553736

[27] Sethuraman N , Jeremiah SS , Ryo A . Interpreting diagnostic tests for SARS‐CoV‐2. JAMA 2020; 323: 2249–2251. 32374370 10.1001/jama.2020.8259

[28] Afzal A . Molecular diagnostic technologies for COVID‐19: limitations and challenges. J Adv Res 2020; 26: 149–159. 32837738 10.1016/j.jare.2020.08.002 PMC7406419

[29] Jayamohan H , Lambert CJ , Sant HJ *et al*. SARS‐CoV‐2 pandemic: a review of molecular diagnostic tools including sample collection and commercial response with associated advantages and limitations. Anal Bioanal Chem 2021; 413: 49–71. 33073312 10.1007/s00216-020-02958-1 PMC7568947

[30] Oran DP , Topol EJ . Prevalence of asymptomatic SARS‐CoV‐2 infection: a narrative review. Ann Intern Med 2020; 173: 362–367. 32491919 10.7326/M20-3012 PMC7281624

[31] Long QX , Tang XJ , Shi QL *et al*. Clinical and immunological assessment of asymptomatic SARS‐CoV‐2 infections. Nat Med 2020; 26: 1200–1204. 32555424 10.1038/s41591-020-0965-6

[32] Feng S , Phillips DJ , White T *et al*. Correlates of protection against symptomatic and asymptomatic SARS‐CoV‐2 infection. Nat Med 2021; 27: 2032–2040. 34588689 10.1038/s41591-021-01540-1 PMC8604724

[33] Hicks SM , Pohl K , Neeman T *et al*. A dual‐antigen enzyme‐linked immunosorbent assay allows the assessment of severe acute respiratory syndrome coronavirus 2 antibody seroprevalence in a low‐transmission setting. J Infect Dis 2021; 223: 10–14. 33009908 10.1093/infdis/jiaa623 PMC7665523

[34] Zhao Q , Lu D , Zhang G , Zhang D , Shi X . Recent improvements in enzyme‐linked immunosorbent assays based on nanomaterials. Talanta 2021; 223: 121722. 33303168 10.1016/j.talanta.2020.121722

[35] Gavor E , Choong YK , Er SY , Sivaraman H , Sivaraman J . Structural basis of SARS‐CoV‐2 and SARS‐CoV antibody interactions. Trends Immunol 2020; 41: 1006–1022. 33041212 10.1016/j.it.2020.09.004 PMC7498231

[36] Bolton JS , Chaudhury S , Dutta S *et al*. Comparison of ELISA With electro‐chemiluminescence technology for the qualitative and quantitative assessment of serological responses to vaccination. Malar J 2020; 19: 159. 32303235 10.1186/s12936-020-03225-5 PMC7165447

[37] Mattiuzzo G , Bentley EM , Hassall M *et al*. Establishment of the WHO international standard and reference panel for anti‐SARS‐CoV‐2 antibody. WHO/BS/2020.2403. Access date: March 08, 2022. World Health Organization. Available from: https://cdn.who.int/media/docs/default‐source/biologicals/ecbs/bs‐2020‐2403‐sars‐cov‐2‐ab‐ik‐17‐nov‐2020_4ef4fdae‐e1ce‐4ba7‐b21a‐d725c68b152b.pdf?sfvrsn=662b46ae_8&download=true

